# Population genetics of *Sida fallax* Walp. (Malvaceae) in the Hawaiian Islands

**DOI:** 10.3389/fpls.2024.1304078

**Published:** 2024-03-01

**Authors:** Mersedeh Pejhanmehr, Michael Benjamin Kantar, Mitsuko Yorkston, Clifford W. Morden

**Affiliations:** ^1^ School of Life Sciences, University of Hawaiʻi, Honolulu, HI, United States; ^2^ Department of Tropical Plant and Soil Sciences, University of Hawaiʻi, Honolulu, HI, United States

**Keywords:** *Sida fallax*, population genetics, Hawaiian Islands, MIG-seq, ecotypes

## Abstract

**Introduction:**

*Sida fallax* (Malvaceae) is the most widespread and variable taxon of Malvaceae in the Hawaiian Islands, growing with a diversity of morphological forms in different habitats including Midway Atoll, Nihoa, and all the main islands. Morphological variation exists within and among populations. The study aimed to investigate the genetic variation within and among populations from various habitats and geographic locations throughout the Hawaiian range of *S. fallax*.

**Methods:**

A total of 124 samples, with up to five samples per population where possible, were collected from 26 populations across six of the main Hawaiian Islands (Kauaʻi, Oʻahu, Maui, Molokaʻi, Lānaʻi, and Hawaiʻi) and Nihoa in the Northwestern Hawaiian Islands. The sampling strategy encompassed collecting populations from different habitats and geographic locations, including coastal and mountain ecotypes, with many intermediate morphological forms. Multiplexed ISSR genotyping by sequencing (MIG-seq) was used to detect single nucleotide polymorphisms (SNP) and genetic differences among individuals and populations were evaluated using PCO analyses.

**Results:**

The relationship of *F_ST_* with the geographical distance between the populations was assessed using the Mantel test. The results showed that populations on a single island were more closely related to each other and to populations on islands within their respective groups than they were to populations on other islands.

**Discussion:**

The overall genetic relationships among islands were, to a large extent, predictive based on island position within the chain and, to a lesser extent, within island topography.

## 1 Introduction

Oceanic archipelagos have long been considered natural laboratories for studying evolution because of their role in speciation and adaptive radiation (Darwin, 1859; Lack, 1947; Carlquist, 1974; Schluter, 2000). The remote archipelago of the Hawaiian Islands (ca. 4000 km from the nearest continental landmass) supports a variety of geographically isolated habitats resulting from the volcanic nature of the islands and ranging from mesic mountain to dry coastal zones. In addition, on the youngest island, lava flows continuously destroy old habitats and create new ones (Sohmer and Gustafson, 1987; Westerbergh and Saura, 1994; Wagner et al., 1999). Furthermore, some plant characteristics can facilitate adaptive radiation. For example, Takayama et al. (2018) suggests that herbaceous perennial herbs with leaky self-incompatible breeding systems, good intra-island dispersal capabilities, and flexible structural and physiological systems are more likely to undergo adaptive radiation in island environments. Importantly, the progenitors of adaptively radiated groups in islands are those that have already been successful in adaptations to different environments in source areas and have undergone eco-geographic speciation. In addition, diversification could be facilitated by ancestral genetic variation and hybridization (Choi et al., 2021). Consequently, the flora of the Hawaiian Islands exhibits some notable cases of adaptive radiation (Wagner and Funk, 1995; Ziegler, 2002; Keeley and Funk, 2011). In many cases, adaptive radiation is accompanied by speciation. Examples include the silversword alliance (Asteraceae; Baldwin and Sanderson, 1998; Carlquist et al., 2003; Landis et al., 2018), Hawaiian *Bidens* (Asteraceae; Knope et al., 2012, 2020) lobeliads (Campanulaceae; Givnish et al., 2009), mints (Lamiaceae; Lindqvist et al., 2003), *Cyrtandra* (Gesneriaceae; Johnson et al., 2019) and *Schiedea* (Caryophyllaceae; Sakai et al., 2006). In these examples, the evolution of widely divergent growth forms and morphologies were derived from a single colonization and are found in a wide range of habitats (Wagner and Funk, 1995; Price and Wagner, 2004). In other cases, adaptive radiation was not followed by abundant speciation and species have highly variable intraspecific morphological diversity. One example is *Metrosideros polymorpha* Gaud., the dominant tree species in the Hawaiian Islands (Percy et al., 2008).

*Sida fallax* (Malvaceae) is widely distributed throughout the Pacific region. It is the most widespread and variable taxon of Malvaceae in the Hawaiian Islands, growing with a diversity of morphological forms and in different habitats of Midway Atoll, Nihoa, and all the main islands. Morphological variation exists both within and among populations, particularly in plant stature, leaf size and shape, degree of pubescence, and inflorescence characters. There are numerous forms that were named for this species in the Hawaiian language recognizing this diversity in plant growth and habitat (Neal, 1948; Handy and Handy, 1991; Krauss, 1993). Ecologically, it is found in a variety of habitats from dry and mesic coastal zones to upland forest habitats, with an elevation range from 0 to 2,000 m (Gagné and Cuddihy, 1990).

**Figure 1:**
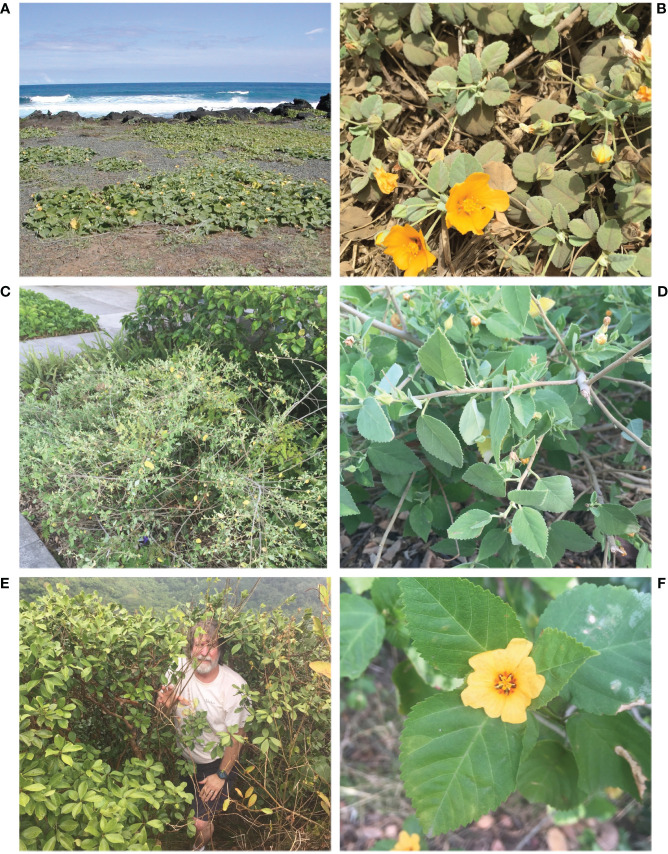
Ecotype variation within *Sida fallax*. Beach ecotype **(A)** habit, **(B)** flower and smaller densely pubescent, ovate leaves; intermediate/inland ecotype **(C)** habit, **(D)** flower buds and mildly pubescent, acute leaves; mountain ecotype **(E)** habit, **(F)** flower and larger glabrous, acute leaves.

There are two ecological forms of *Sida fallax* with many intermediates in the Hawaiian Islands (**Figure 1**) (Wagner et al., 1999; Yorkston and Daehler, 2006). A low elevation ecotype occurs along beaches and in dry, coastal shrublands. These are low sprawling shrubs that are typically less than 50 cm tall with small (up to 4-cm long), densely pubescent ovate leaves. This form can take the extreme form of a prostrate subshrub in coastal communities. *Sida fallax* from other Pacific locations exhibit this form only. In contrast, the mountain ecotype is an erect shrub that typically reaches 1–1.5 m in height (in some cases exceeding 2 m) with large (commonly 4 to 8 cm long), glabrous or nearly glabrous leaves. These forms are found in upland communities and mesic forest sites, usually away from coastal influences (Wagner et al., 1999; Yorkston and Daehler, 2006). The many intermediate morphological types between these extreme forms, many of which were recognized by early Hawaiians (see above), occur in a variety of habitats throughout the Hawaiian Islands.

Several studies have been carried out on the different ecotypes of *S. fallax*. Stephens (2000) compared the ecophysiology of beach and mountain ecotypes and found that the beach ecotype had a greater tolerance of drought than the mountain ecotype. Experimental crosses carried out by Yorkston and Daehler (2006) between the beach and mountain forms demonstrated a lack of fertility barriers between them. Triplett et al. (2024) observed that coastal leaves of *S. fallax* exhibited a greater photosynthetic advantage from amphistomy compared to nearby montane leaves in more closed forest environments. Furthermore, a phylogenetic study of all ecotypes of *S. fallax* from the Hawaiian Islands and areas throughout the Pacific supported it as a single species with very little variation among the nuclear and chloroplast gene regions examined (Pejhanmehr, 2022; Pejhanmehr et al., 2022). Examples of species ecotypes that are genetically different are known (Korecký et al., 2021).The objective of the present study was to investigate the genetic variation within and among populations from the various habitats and geographic locations throughout the Hawaiian range of *S. fallax*. The various ecological and morphological forms of *S. fallax* discussed above occur on many of the islands. However, the expectation is that genetic relations will mirror the dispersion among islands. Parallel evolution is occurring with morphologically different forms on a single island being more closely related to each other than they are to morphologically similar forms on different islands.

## 2 Materials and methods

### 2.1 Sampling and DNA extraction

**Figure 2:**
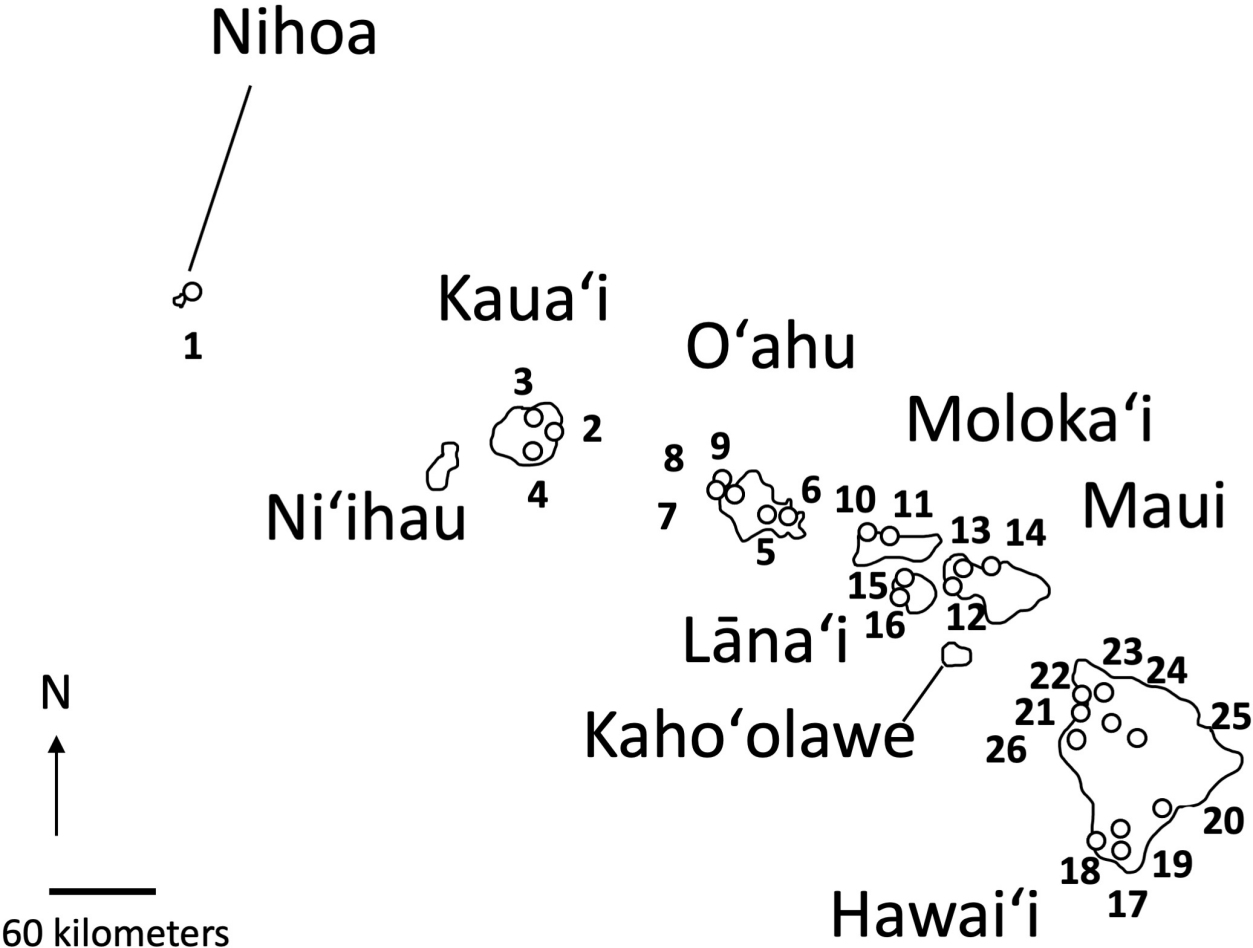
Location of the 26 populations of *Sida fallax* on Nihoa and the main Hawaiian Islands. Numbers indicate the populations identified in **Table 1**.

When possible, up to five samples were selected from collections representing 26 distinct populations of *Sida fallax* throughout the Hawaiian Islands. For some populations, fewer samples were available. Collections represented widely separated populations from the coastal strand to montane zones and habitats ecologically intermediate between these two extremes. Similarly, collections within populations were made from widely spaced individuals. Ecotypes were assigned based on proximity to coastal communities and elevation, and were identified as either “beach” or “mountain/inland”. Populations were from six of the main Hawaiian Islands (Kauaʻi, Oʻahu, Maui, Molokaʻi, Lānaʻi, and Hawaiʻi) and Nihoa in the Northwestern Hawaiian Islands (**Figure 2**; **Table 1**). Vouchers were deposited in the Joseph F. Rock (HAW) herbarium of the University of Hawaiʻi at Mānoa. Total genomic DNA was extracted using the CTAB method of Doyle and Doyle (1987) with some modifications (Morden et al., 1996) and deposited in the Hawaiian Plant DNA Library (HPDL) at the University of Hawaiʻi at Mānoa.

**Table 1:** Taxa used for *Sida fallax* population genetic analyses with population number, voucher identification, Hawaiian Plant DNA Library accession number (HPDL), collection locality, and ecotype.

Pop No.	Voucher identification	HPDL	Origin	Latitude and Longitude	Ecotype
1	*Caraway s.n.* (HAW)	10577–10583	Middle ridge, Nihoa, Hawaiian Islands	23° 20’ 15.54’’ N”, 161°55’18.57”W	Mountain/inland
2	*Adams 262* (PTBG)	9615, 9616	Makaleha, Kauaʻi, Hawaiian Islands	22° 7’12.00”N, 159°25’12.00”W	Mountain/inland
3	*Adams s.n.* (PTBG)	8319–8321	Nukoliʻi beach park, Kauaʻi, Hawaiian Islands	22° 0’27.02”N, 159°20’22.52”W	Beach
4	*Adams 261* (PTBG)	9617–9621	Mahaʻulepu, Kauaʻi, Hawaiian Islands	21°52’29.03”N, 159°26’17.74”W	Beach
5	*Pejhanmehr 101* (HAW)	8278, 8290, 8291, 8293, 8294	Waʻahila Ridge, Oʻahu, Hawaiian Islands	21°18’26.18”N,157°47’56.21”W	Mountain/inland
6	*Pejhanmehr 102* (HAW)	8303, 8310–8313	Makapuʻu point, eastside near highway and near sandy beach Oʻahu, Hawaiian Islands	21°18’39.64”N,157°39’37.03”W	Beach
7	*Pejhanmehr 103* (HAW)	10075, 10080, 10086–10088	Kaʻena Point, Near Parking, Oʻahu, Hawaiian Islands	21°34’27.60”N,158°16’47.05”W	Beach
8	*Pejhanmehr 104* (HAW)	10089, 10091, 10094–10096	Kaʻena Point, After Iron Wood Bush, Oʻahu, Hawaiian Islands	21°34’27.96”N,158°16’42.73”W	Mountain/inland
9	*Pejhanmehr 105* (HAW)	10121, 10127–10130	Kealia Trail, Oʻahu, Hawaiian Islands	21°34’19.37”N,158°12’35.80”W	Mountain/inland
10	*Morden s.n.* (HAW)	8339–8343	Moʻomomi dunes, Molokaʻi, Hawaiian Islands	21°12’2.77”N, 157° 9’32.87”W	Beach
11	*Morden s.n.* (HAW)	8588–8592	Anahaki upper Moʻomomomi, Molokaʻi, Hawaiian Islands	21°11’53.53”N, 157° 7’43.96”W	Mountain/inland
12	*Morden s.n.* (HAW)	8323–8327	Olowalu, above stream, Maui, Hawaiian Islands	20°49’28.15”N, 156°37’18.44”W	Beach
13	*Morden s.n.* (HAW)	8328, 8334–8337	Keʻopuolani Park on top of the sand dunes, Maui, Hawaiian Islands	20°53’38.80”N, 156°29’0.16”W	Beach
14	*Baldos s.n.* (HAW)	10554	Kanaha Beach Park, Maui, Hawaiian Islands	20°53’58.93”N, 156°26’26.12”W	Beach
15	*Bogner 101* (HAW)	10558,10560–10563	Near Keomoku Road, Lānaʻi, Hawaiian Islands	20.88301° N, 156.89902° W	Mountain/inland
16	*Bogner 102* (HAW)	10570–10574	Palawai Basins, Lānaʻi, Hawaiian Islands	20.76977° N, 156.90915° W	Mountain/inland
17	*Baldos s.n.* (HAW)	10543–10547	South Point, Hawai’i, Hawaiian Islands	18°54’49.10”N, 155°40’59.96”W	Beach
18	*Morden 2511* (HAW)	10174, 10178, 10181, 10182, 10186	Punaluʻu Beach, Hawai’i, Hawaiian Islands	19°07’57.3”N, 155°30’21.3”W	Beach
19	*Morden 2514* (HAW)	10236–10239, 10241	Upper South Point, at coastline, Hawaiʻi, Hawaiian Islands	18°55’00.9”N, 155°40’06.4”W	Beach
20	*Morden 2512* (HAW)	10211,10212, 10215,10196, 10201	Upper South Point, above coast, Hawaiʻi, Hawaiian Islands	18°55’13.5”N, 155°40’33.1”W	Mountain/inland
21	*Pejhanmehr 106* (HAW)	10606, 10613, 10617–10619	Waikōloa, along highway, Hawaiʻi, Hawaiian Islands	19°57’12.0”N, 155°49’40.6”W	Mountain/inland
22	*Pejhanmehr 107* (HAW)	10622, 10628, 10636–10638	Kawaihae near Heidi, Hawaiʻi, Hawaiian Islands	20°01’37.4”N, 155°49’20.4”W	Beach
23	*Pejhanmehr 108* (HAW)	10645, 10656, 10657, 10660, 10668	Koai’a Tree Sanctuary, Hawaiʻi, Hawaiian Islands	20°02’54.0”N, 155°44’12.7”W	Mountain/inland
24	*Pejhanmehr 109* (HAW)	10590–10594	Saddle Road, below Waikiʻi, Hawaiʻi, Hawaiian Islands	19°53’06.5”N, 155°40’06.3”W	Mountain/inland
25	*Baldos s.n.* (HAW)	10550–10553	Saddle Road, Hawaiʻi, Hawaiian Islands	19°43’49.19”N, 155°30’8.80”W	Mountain/inland
26	*Pejhanmehr 110* (HAW)	10677–10679, 10696, 10697	Belt Road, between Kailua and Waikōloa, Hawaiʻi, Hawaiian Islands	19°46’42.8” N, 155°54’45.0” W	Mountain/inland

Herbarium acronyms follow Thiers (2021).

### 2.2 MIG-seq library construction and sequencing

In total, 124 samples from 26 populations (**Figure 2**; **Table 1**) were selected for Multiplexed ISSR genotyping by sequencing (MIG-seq) to detect single nucleotide polymorphisms (SNP) (Suyama and Matsuki, 2015). MIG-seq library preparation and primer selection were performed according to Suyama and Matsuki (2015). After measurement of the final concentration of the libraries, approximately 10 pM of the libraries were used for sequencing on an Illumina MiSeq Sequencer (Illumina, San Diego, California), using a MiSeq Reagent Kit v3 (150 cycle, Illumina).

### 2.3 Quality control and SNP detection

Adapter and anchor sequence trimming of raw data were performed according to Suyama and Matsuki (2015), using FASTQ Trimmer (Galaxy Version 1.1.5) (Blankenberg et al., 2010; Afgan et al., 2018). High-quality reads were assessed using the Filter by Quality application (Galaxy Version 1.0.2+galaxy0) (Gordon, 2010; Afgan et al., 2018) with the criterion of q = 30 and p = 40 (q: quality cut-off value, p: percent of bases in a sequence that must have quality equal to or higher than q). Cutadapt (Galaxy Version 3.4+galaxy0) (Martin, 2011; Afgan et al., 2018) was used to remove reads from extremely short library entries (Maximum error rate = 0.01). Filtered reads were assembled using *de novo* map pipelines (ustacks, cstacks, sstacks, gstacks) in Stacks2: *de novo* map (Galaxy Version 2.4+galaxy1) (Catchen et al., 2011, Catchen et al., 2013; Afgan et al., 2018) with the following settings: Mismatches allowed between loci when processing a single individual = 2, Mismatches allowed between loci when building the catalog = 1, minimum percentage of individuals in a population required to process a locus for that population = 0, and minimum number of populations a locus must be present in to process a locus = 1.

### 2.4 Population genetic analysis

The assembled reads (gstackes) were used as input for population genetic analysis using Stacks2: populations (Galaxy Version 2.4+galaxy1) (Catchen et al., 2011, Catchen et al., 2013; Afgan et al., 2018) with the following settings: Minimum percentage of individuals in a population required to process a locus for that population = 0, Minimum number of populations a locus must be present in to process a locus = 1, minimum percentage of individuals across populations required to process a locus = 0, Fraction of samples possessing both loci to prune remaining samples from analysis = 1. Robustness of results was examined by choosing different numbers for Minimum percentage of individuals in a population required to process a locus for that population, and minimum percentage of individuals across populations required to process a locus, i.e., 0 and 0.7, 0.5 and 0, 0.7 and 0, 0 and 0.5, 0.4 and 0, 0.3 and 0.1, 0.45 and 0, 0.3 and 0, 0.1 and 0, and 0.2 and 0. The outputs of Stacks2: populations were used as input data to generate covariance values from population allele frequencies using GenoDive v2.0b27 (Meirmans and Van Tienderen, 2004) to investigate relationships of individuals within and among populations. Genetic similarity indices were calculated using both Gower (1971) and Nei and Li (1979) similarity coefficients for populations using MVSP Plus ver. 3.1 (Kovach, 2007). Relationships within and among populations were estimated from the similarity matrixes using principal coordinate analysis (PCO) with MVSP Plus ver. 3.1 (Kovach, 2007) using Gower similarity (Gower, 1971). The outputs of Stacks2: populations that included the SNP sites of all the samples was converted to VCF format and used to reconstruct a neighbor joining tree in VCF2PopTree (**Supplementary Figure 1**) (Subramanian et al., 2019). The relationship of *F_ST_* with the geographical distance between the populations was assessed using the function mantel.test in ade4 library of the R package ape v5.3 (Dray and Dufour, 2007; R Core Team, 2021) with 9,999 permutations. Finally, a scatter plot was created using ggplot2 function in R (Wickham, 2016) to visualize the relationship between *F_ST_* values and the distances between paired populations and ecotypes.

## 3 Results

### 3.1 Numbers of reads, SNPs, and polymorphic loci

The number of reads per sample ranged from 1,789 to 216,730 in the raw data (from 1,539 to 210,631 after quality filtering) and the removal rate varied from 0 to 11% for 124 individuals. The forward and reverse reads were treated independently because they do not overlap and could not be combined. The results of both reads were compared and provided near-identical outcomes, and only the reverse reads are presented here. Stacks verified 57,475 loci composed of 3,838,616 (bp) sites. Twenty-five sites that were pseudo-variant (PCR error) loci and detected only in one sample were filtered (Suyama and Matsuki, 2015), and 12,796 variant sites (or SNP) remained. The mean genotyped sites per locus was 66.79 bp (stderr 0.01).

The population diversity statistics are summarized in **Table 2**. Polymorphic sites per population ranged from 82 to 1209 and variant sites (SNP) from 999 to 6074. Private alleles (alleles occurring in only a single population) ranged from 50 to 414 among populations. Observed heterozygosity ranged from 0.082 to 0.136 and nucleotide diversity (Pi) from 0.082 to 0.131. Among all population diversity statistics, the smallest values belonged to population 14 from Maui Beach where only one individual was found. The largest values for each statistic were mostly from Hawaiʻi Island (populations 17 and 24, mountain/inland habitats) and Lānaʻi (population 21, beach habitat). The inbreeding coefficient (*F_IS_*) ranged from -0.015 on Nihoa (population 1, mountain/inland habitat of Nihoa) to 0.009 on both Kauaʻi (population 4, beach habitat) and Hawaiʻi (population 17, beach habitat).The average value of observed heterozygosity comparison between beach (0.109) and mountain/inland (0.111) habits were similar.

**Table 2:** Population diversity statistics.

Pop ID	All	Pm_s	Variant	Private	Var	StdErr	Obs_Het	StdErr	Obs_Hom	StdErr	Exp_Het	StdErr	Exp_Hom	StdErr	Pi	StdErr	Fis	StdErr
1	571725	856	5476	263	5.051	0.030	0.111	0.004	0.889	0.004	0.068	0.002	0.932	0.002	0.102	0.004	-0.015	0.030
2	402447	383	3906	182	0.243	0.008	0.088	0.004	0.912	0.004	0.047	0.002	0.953	0.002	0.083	0.004	-0.007	0.008
3	524893	608	4838	212	0.574	0.011	0.100	0.004	0.900	0.004	0.058	0.002	0.942	0.002	0.100	0.004	-0.001	0.011
4	409642	432	3631	227	1.045	0.017	0.082	0.004	0.918	0.004	0.053	0.002	0.947	0.002	0.088	0.004	0.009	0.017
5	592719	641	4823	373	1.349	0.017	0.082	0.004	0.918	0.004	0.057	0.002	0.943	0.002	0.087	0.003	0.006	0.017
6	705392	956	5951	399	1.815	0.017	0.108	0.004	0.892	0.004	0.070	0.002	0.930	0.002	0.111	0.004	0.005	0.017
7	514824	631	4446	339	0.908	0.014	0.101	0.004	0.899	0.004	0.063	0.002	0.937	0.002	0.104	0.004	0.005	0.014
8	618086	834	5407	326	1.969	0.019	0.103	0.004	0.897	0.004	0.066	0.002	0.934	0.002	0.102	0.004	-0.002	0.019
9	599487	809	5438	255	2.260	0.020	0.107	0.004	0.893	0.004	0.066	0.002	0.934	0.002	0.103	0.004	-0.006	0.020
10	629118	1049	5538	414	2.240	0.020	0.129	0.004	0.871	0.004	0.080	0.002	0.920	0.002	0.124	0.004	-0.010	0.020
11	526591	727	4738	306	1.453	0.018	0.108	0.004	0.892	0.004	0.068	0.002	0.932	0.002	0.111	0.004	0.004	0.018
12	501152	627	4645	272	1.004	0.015	0.100	0.004	0.900	0.004	0.061	0.002	0.939	0.002	0.098	0.004	-0.004	0.015
13	587089	1015	5455	337	2.333	0.021	0.129	0.004	0.871	0.004	0.081	0.002	0.919	0.002	0.126	0.004	-0.003	0.021
14	116124	82	999	50	0.000	0.000	0.082	0.009	0.918	0.009	0.041	0.004	0.959	0.004	0.082	0.009	0.000	0.000
15	591205	976	5580	323	2.395	0.021	0.119	0.004	0.881	0.004	0.074	0.002	0.926	0.002	0.113	0.004	-0.011	0.021
16	654765	1072	5962	412	2.332	0.020	0.120	0.004	0.880	0.004	0.075	0.002	0.925	0.002	0.116	0.004	-0.007	0.020
17	706318	616	4323	244	1.072	0.016	0.096	0.004	0.904	0.004	0.064	0.002	0.936	0.002	0.102	0.004	0.009	0.016
18	545314	892	5236	246	2.277	0.021	0.121	0.004	0.879	0.004	0.074	0.002	0.926	0.002	0.115	0.004	-0.009	0.021
19	656137	1061	5931	311	2.020	0.018	0.132	0.004	0.868	0.004	0.079	0.002	0.921	0.002	0.127	0.004	-0.007	0.018
20	485995	776	4893	130	2.185	0.021	0.116	0.004	0.884	0.004	0.070	0.002	0.930	0.002	0.108	0.004	-0.014	0.021
21	671176	1209	6074	373	2.412	0.020	0.130	0.004	0.870	0.004	0.083	0.002	0.917	0.002	0.125	0.004	-0.007	0.020
22	665764	1085	5910	387	2.062	0.019	0.126	0.004	0.874	0.004	0.079	0.002	0.921	0.002	0.125	0.004	0.000	0.019
23	643946	1006	5641	350	1.742	0.018	0.126	0.004	0.874	0.004	0.078	0.002	0.922	0.002	0.124	0.004	-0.002	0.018
24	669847	1164	6036	334	2.345	0.020	0.136	0.004	0.864	0.004	0.085	0.002	0.915	0.002	0.131	0.004	-0.008	0.020
25	470827	553	4172	197	0.594	0.012	0.098	0.004	0.902	0.004	0.061	0.002	0.939	0.002	0.101	0.004	0.004	0.012
26	298318	384	3151	77	0.472	0.012	0.116	0.006	0.884	0.006	0.060	0.003	0.940	0.003	0.113	0.006	-0.004	0.012
Beach ave.	546813.917	754.5	4741.917	286.5	1.446	0.741	0.109	0.017	0.891	0.017	0.067	0.012	0.933	0.012	0.109	0.015	-0.001	0.006
Mountain/inland ave.	556938.143	813.571	5092.643	278.643	1.914	1.136	0.111	0.015	0.889	0.015	0.068	0.010	0.932	0.010	0.109	0.013	-0.005	0.006

All, all sites. Pm_s, polymorphic sites (when different individuals differ from the reference stack at the same position); Variant, variant sites (SNP); Private, private alleles; alleles that occur in only that population; Var, number of variances per population.; StdErr, standard error; Obs_Het, observed heterozygosity; Exp_Het, expected heterozygosity; Obs_Hom, observed homozygosity; Exp_Hom, expected homozygosity; Pi, nucleotide diversity (number of nucleotide differences per site between two randomly chosen sequences from a population).; F_IS_, inbreeding coefficient.

### 3.2 Population differentiation and genetic distance

The Mantel test identified a significant positive correlation between genetic and geographic distances among *S. fallax* populations (r=0.295, p = 0.0209). Populations that were more closely associated geographically were also more closely related genetically. Overall, most *F_ST_* values were approximately 0.20 with very low genetic variance among the 26 populations (**Table 3**). However, populations that were located on the same island and in similar habitats had smaller *F_ST_* values, and populations on different islands and in different habitats had larger *F_ST_* values. The smallest pairwise *F_ST_* was between two populations from the mountain/inland habitat of Hawaiʻi Island (populations 21 and 26) that were located close to each other (*F_ST_* = 0.15). In contrast, populations from different habitats and islands showed the largest *F_ST_*, for example population 2 from a mountain/inland habitat on Kauaʻi and population 14 from a beach habitat on Maui (*F_ST_* = 0.44). The scatter plot also indicates a significant positive correlation between genetic and geographic distances among *S. fallax* populations (**Supplementary Figure 2**). Additionally, it shows that beach-to-beach population pairs have a higher average *F_ST_* than mountain/inland paired populations.

**Table 3:** Pairwise F_ST_ among 26 population of Sida fallax in Hawaiian Islands.

	1	2	3	4	5	6	7	8	9	10	11	12	13	14	15	16	17	18	19	20	21	22	23	24	25	26
**1**		**0.27**	**0.23**	**0.25**	**0.27**	**0.20**	**0.23**	**0.21**	**0.22**	**0.21**	**0.24**	**0.26**	**0.22**	**0.35**	**0.22**	**0.21**	**0.28**	**0.24**	**0.26**	**0.24**	**0.22**	**0.26**	**0.25**	**0.24**	**0.26**	**0.26**
**2**			**0.31**	**0.35**	**0.32**	**0.30**	**0.32**	**0.28**	**0.30**	**0.26**	**0.32**	**0.34**	**0.28**	**0.44**	**0.27**	**0.27**	**0.35**	**0.29**	**0.32**	**0.28**	**0.27**	**0.29**	**0.29**	**0.29**	**0.34**	**0.35**
**3**				**0.26**	**0.28**	**0.24**	**0.24**	**0.23**	**0.24**	**0.21**	**0.24**	**0.27**	**0.22**	**0.36**	**0.22**	**0.22**	**0.29**	**0.25**	**0.28**	**0.25**	**0.24**	**0.23**	**0.25**	**0.25**	**0.28**	**0.27**
**4**					**0.31**	**0.27**	**0.28**	**0.27**	**0.27**	**0.25**	**0.28**	**0.31**	**0.25**	**0.38**	**0.25**	**0.27**	**0.34**	**0.28**	**0.30**	**0.28**	**0.28**	**0.27**	**0.28**	**0.28**	**0.30**	**0.32**
**5**						**0.27**	**0.28**	**0.24**	**0.25**	**0.24**	**0.26**	**0.28**	**0.24**	**0.32**	**0.23**	**0.25**	**0.31**	**0.27**	**0.29**	**0.25**	**0.26**	**0.28**	**0.27**	**0.28**	**0.29**	**0.27**
**6**							**0.23**	**0.21**	**0.23**	**0.22**	**0.24**	**0.26**	**0.23**	**0.33**	**0.21**	**0.23**	**0.28**	**0.24**	**0.27**	**0.24**	**0.24**	**0.26**	**0.25**	**0.25**	**0.28**	**0.25**
**7**								**0.22**	**0.23**	**0.22**	**0.26**	**0.29**	**0.23**	**0.35**	**0.22**	**0.22**	**0.29**	**0.25**	**0.28**	**0.24**	**0.24**	**0.25**	**0.26**	**0.25**	**0.27**	**0.26**
**8**									**0.21**	**0.20**	**0.23**	**0.25**	**0.21**	**0.35**	**0.20**	**0.21**	**0.27**	**0.23**	**0.25**	**0.22**	**0.22**	**0.24**	**0.24**	**0.24**	**0.25**	**0.26**
**9**										**0.20**	**0.24**	**0.27**	**0.22**	**0.33**	**0.20**	**0.22**	**0.29**	**0.25**	**0.26**	**0.24**	**0.23**	**0.23**	**0.25**	**0.25**	**0.28**	**0.26**
**10**											**0.19**	**0.21**	**0.19**	**0.27**	**0.19**	**0.18**	**0.23**	**0.21**	**0.23**	**0.18**	**0.19**	**0.21**	**0.21**	**0.21**	**0.22**	**0.20**
**11**												**0.26**	**0.20**	**0.29**	**0.21**	**0.20**	**0.25**	**0.22**	**0.25**	**0.20**	**0.21**	**0.24**	**0.24**	**0.23**	**0.24**	**0.22**
**12**													**0.22**	**0.31**	**0.21**	**0.21**	**0.26**	**0.22**	**0.25**	**0.22**	**0.21**	**0.23**	**0.24**	**0.24**	**0.27**	**0.25**
**13**														**0.27**	**0.18**	**0.17**	**0.24**	**0.21**	**0.21**	**0.20**	**0.19**	**0.20**	**0.21**	**0.20**	**0.23**	**0.19**
**14**															**0.25**	**0.28**	**0.29**	**0.22**	**0.30**	**0.26**	**0.27**	**0.30**	**0.27**	**0.25**	**0.30**	**0.36**
**15**																**0.18**	**0.22**	**0.20**	**0.20**	**0.18**	**0.17**	**0.20**	**0.20**	**0.20**	**0.22**	**0.20**
**16**																	**0.24**	**0.20**	**0.22**	**0.18**	**0.18**	**0.21**	**0.20**	**0.21**	**0.22**	**0.19**
**17**																		**0.19**	**0.23**	**0.20**	**0.21**	**0.24**	**0.24**	**0.23**	**0.28**	**0.20**
**18**																			**0.19**	**0.16**	**0.18**	**0.20**	**0.20**	**0.21**	**0.21**	**0.20**
**19**																				**0.16**	**0.18**	**0.21**	**0.22**	**0.21**	**0.24**	**0.20**
**20**																					**0.16**	**0.19**	**0.17**	**0.18**	**0.19**	**0.18**
**21**																						**0.17**	**0.18**	**0.17**	**0.20**	**0.15**
**22**																							**0.20**	**0.20**	**0.23**	**0.18**
**23**																								**0.18**	**0.23**	**0.20**
**24**																									**0.21**	**0.19**
**25**																										**0.22**

### 3.3 Genetic Assignment

**Figure 3:**
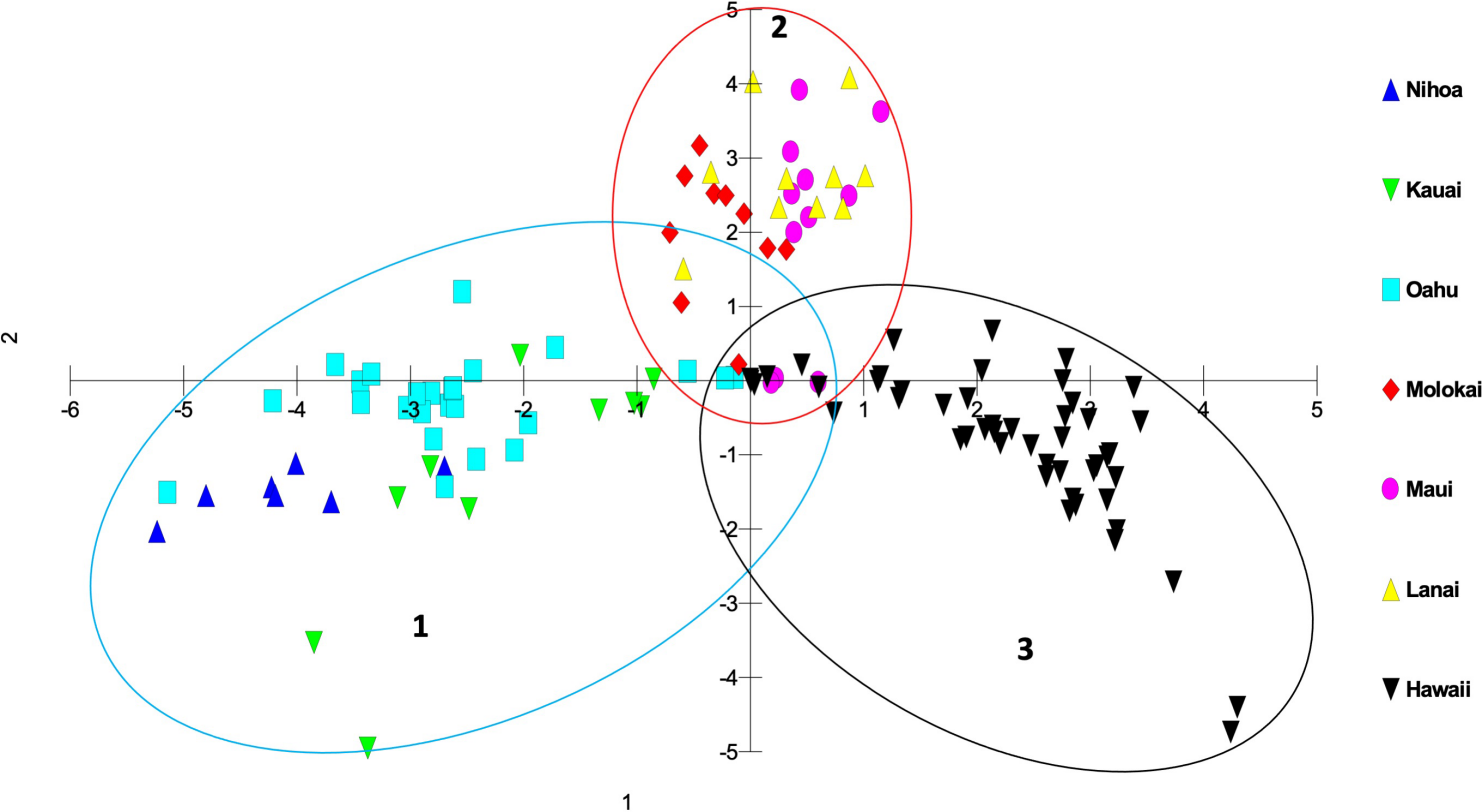
Principal coordinate analysis using MIG-seq data identifying individuals from each island in the Hawaiian Islands using a matrix of covariance values calculated from population allele frequencies. The three groups of *S. fallax* populations include 1) Nihoa, Oʻahu and Kauaʻi; 2) Maui, Molokaʻi, and Lānaʻi (Maui Nui); 3) Hawaiʻi.

**Figure 4:**
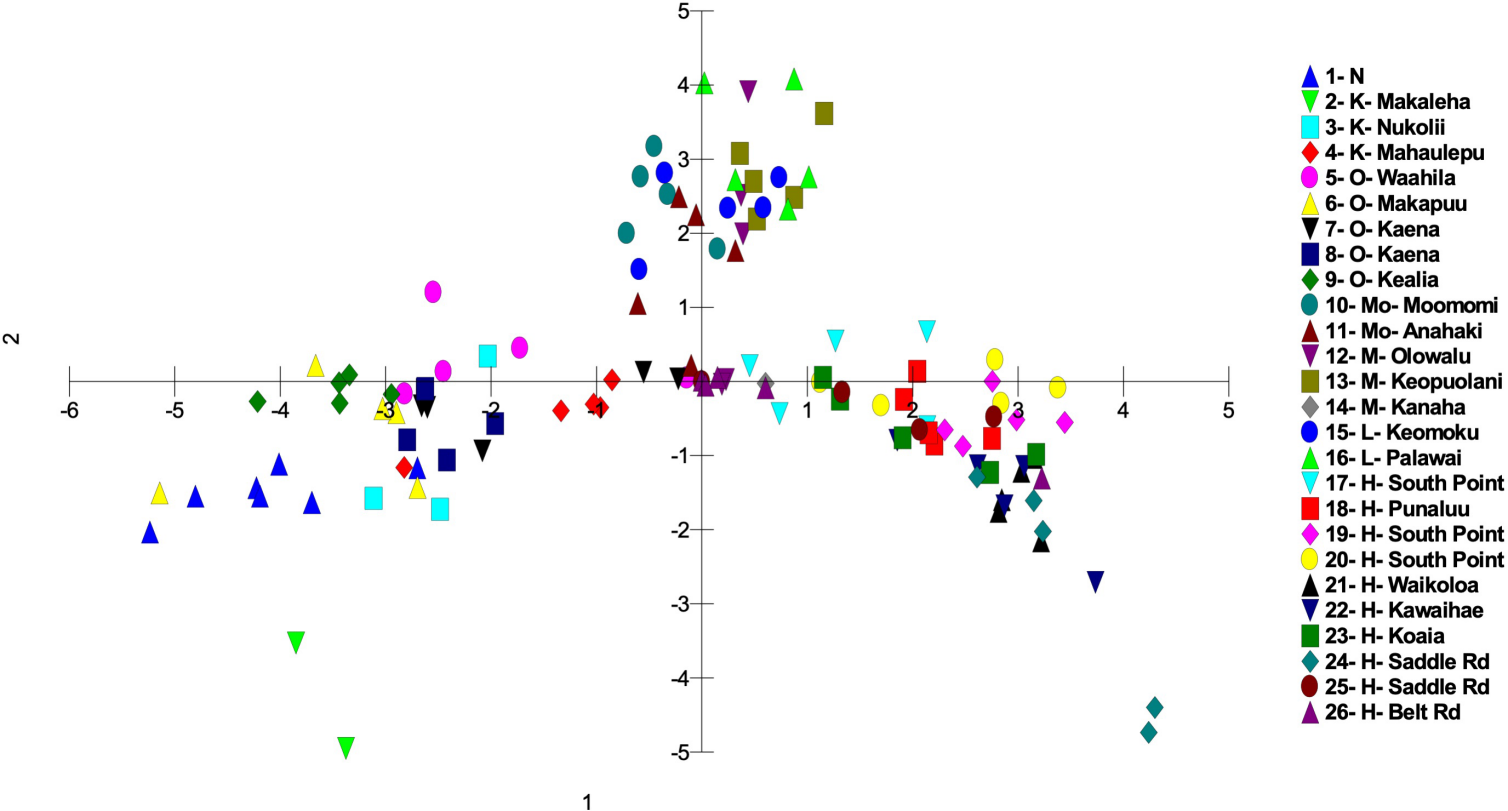
Principal coordinate analysis using MIG-seq data identifying the 26 *S. fallax* populations in across the Hawaiian Islands using a matrix of covariance values calculated from population allele frequencies. Island abbreviations: N, Nihoa; K, Kauaʻi; O, Oʻahu; M, Maui; Mo, Molokaʻi; L, Lānaʻi; H, Hawaiʻi (**Table 1**). Population numbers correspond to those in **Figure 2**.

Genetic differences among individuals and populations from across the range of habitat and locations of *S. fallax* in the Hawaiian Islands were evaluated using PCO analyses. Three main islands groupings were evident: 1) Oʻahu, Kauaʻi and Nihoa; 2) Maui, Molokaʻi, and Lānaʻi (collectively referred to as Maui Nui); 3) Hawaiʻi Island. Populations from each island grouping intersect at the center of the graph (**Figures 3**, **4**). The phylogenetic analysis indicated the same island grouping as well (**Supplementary Figure 1**).

**Figure 5:**
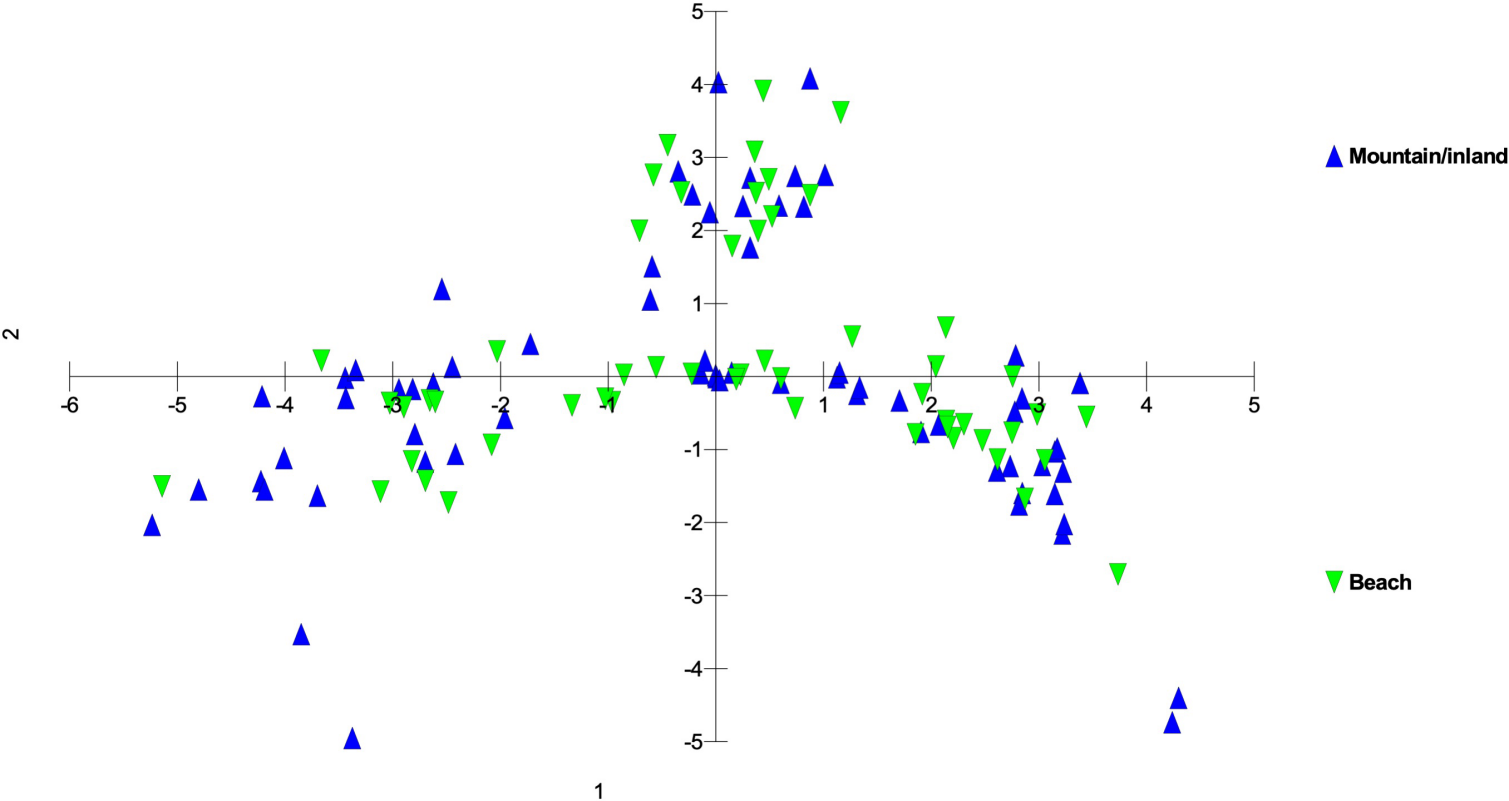
Principal coordinate analysis using MIG-seq data of *S. fallax* populations in Hawaiian Islands distinguished by habitat - mountain or inland vs. beach ecotypes - based on the same analysis presented in **Figure 3**. Nihoa plants, although near coastal were considered inland as there is no beach habitat on that island.

There was a trend of coastal/beach populations occurring more predominantly near the zone of intersection (ZOI) of the island groups and the mountain/inland or most isolated populations being more distant from this zone of intersection (**Figure 5**). Within the Oʻahu-Kauaʻi-Nihoa group, populations near the ZOI include the coastal Kaʻena Point, Oʻahu (population 7), and Mahaʻulepu, Kauaʻi (Population 4) populations. One individual from Waʻahila (a mountain population; population 5) was located within the ZOI, but the remaining individuals from this population were not. Other populations outside the ZOI included those from Makaleha and Nukoliʻi from Kauaʻi; and Kealia, inland Kaʻena, and Makapuʻu from Oʻahu. Plants from Nihoa (the island being well separated from the main Hawaiian Islands) were well differentiated from the ZOI (**Figure 2**). The habitat of Nihoa is largely mountain basaltic slopes with no beach habitat.

The second island group consisted of populations from Maui Nui. All Maui Nui populations were collected from beaches or inland regions close to the beach. A few individuals from Molokaʻi (population 11) were within the ZOI (**Figure 4**), but most fell outside it. Maui Nui plants form a cohesive group that does not exhibit extensive diversification and does not extend as far from the ZOI as do the other two groups.

The third island group consisted of plants from Hawaiʻi Island. As the largest island, more populations (10) were investigated, throughout the island, than on the other islands. Populations extend from the ZOI, with plants from South Point and Belt Road (populations 17 and 26, respectively) relatively close to the ZOI to populations well separated from the ZOI, notably those from Saddle Road (population 24). There was broad overlap in coastal/beach and mountain/inland populations within the diversity of the Hawaiʻi Island group.

## 4 Discussion

It is common in the analysis of population variation among species that are distributed throughout the Hawaiian Islands that individuals and populations within an island show a closer genetic relationship to other populations on that island than to populations from other islands. This has been demonstrated repeatedly using other methods for a wide variety of genera including *Hibiscus* (Malvaceae; Huppman, 2013), *Erythrina* (Fabaceae; Grave, 2018), and *Sesbania* (Fabaceae; Cole and Morden, 2021). In this study of *Sida fallax*, the population organization reflected a Nihoa-Kauaʻi-Oʻahu group, a Maui Nui group, and a Hawaiʻi Island group. Populations within each of these three groups broadly overlapped genetically and were distinct from those in the other two groups, suggesting that each group has retained intra-group connectivity. On closer inspection of the variation in the previous studies noted above it is evident that, although the island populations are distinct, they also are consistent with these same three groups of islands found for *Sida fallax*. A major distinction in this study is that the three groups are connected (the ZOI) suggesting gene flow still exists among them. This is probably a reflection of *Sida fallax* having many coastal populations and fruits with indehiscent mericarps capable of long-distance dispersal via ocean currents. These factors allow it to maintain genetic cohesion among populations throughout the Hawaiian Islands and even across the Pacific Ocean (Pejhanmehr, 2022; Pejhanmehr et al., 2022) as has been found in other groups (Baum et al., 2004; Takayama et al., 2008; Areces-Berazain and Ackerman, 2016; Areces-Berazain and Ackerman, 2017).

Within each of the three island groups, populations were largely intermixed genetically and there was no island-by-island separation. There was no clear genetic delineation between beach and mountain/inland ecotypes. However, there was some evidence of more coastal or beach populations being closer to the zone of intersection and inland populations more distant from it, suggesting diversification within each of these groups. However, there was also a large degree of overlap suggesting continuing introgression among the forms of *Sida fallax* as well as across the habitats. As found by Yorkston and Daehler (2006), there are no fertility barriers among populations and forms of the species. *Sida fallax* are pollinated by native insects such as yellow-faced bees, *Hylaeus* spp., and non-native insects such as honeybees, *Apis mellifera*, and other generalist Hymenoptera, butterflies, and moths (Yorkston and Daehler, 2006; Shay et al., 2016; Shay and Drake, 2018; Aslan et al., 2019). Fruits of other *Sida* spp. were demonstrated to disperse readily by air or water when mature (Raju and Rani, 2016).

Some populations fell far from the zone of intersection. These included the Nihoa, Makaleha (Kauaʻi), and Saddle Road (Hawaiʻi) mountain ecotype populations, along with some individuals from beach ecotype populations. The mountain ecotypes were more ecologically isolated from other parts of islands so they may have some unique genetic differences. Moreover, the beach ecotypes could receive seeds from other parts of the Pacific via ocean currents and accumulate some unique genetic diversity. This suggests that there is ongoing population diversification, but gene flow among populations is keeping them from fully segregating from others within their groups.

Overall, populations on a single island were more closely related to each other and to populations on islands within their respective groups than they were to populations on other islands. Due to the higher probability of long-distance seed dispersal via ocean currents for beach ecotype populations, they exhibit somewhat higher genetic diversity among themselves rather than mountain ecotype populations that are more isolated (**Supplementary Figure 2**). The overall genetic relationships among islands were to a large extent predictive based on island position within the chain, and, to a lesser extent, within island topography.

## Data availability statement

The datasets presented in this study can be found in online repositories. The names of the repository/repositories and accession number(s) can be found below: https://www.ncbi.nlm.nih.gov/, PRJNA979219.

## Ethics statement

Written informed consent was obtained from the individual(s) for the publication of any identifiable images or data included in this article.

## Author contributions

MP: Conceptualization, Data curation, Investigation, Writing – original draft, Writing – review & editing. MK: Conceptualization, Formal analysis, Writing – review & editing. MY: Formal analysis, Methodology, Writing – review & editing. CM: Conceptualization, Funding acquisition, Investigation, Project administration, Resources, Supervision, Writing – review & editing.

## Supplementary material

Supplementary Figure 1 Neighbor Joining tree using MIG-seq data for 124 individuals in 26 populations of *Sida fallax* across the Hawaiian Islands. Island abbreviations: N, Nihoa; K, Kauaʻi; O, Oʻahu; M, Maui; Mo, Molokaʻi; L, Lānaʻi; H, Hawaiʻi. Population numbers correspond to those in **Table 1**. Blue color: mountain/inland populations; Green color: beach populations.

Supplementary Figure 2 Scatter plot showing *F_ST_* values versus distances between paired populations. Mtn: mountain.
