# Supplementary figures and images for: Population genetics of Sida fallax Walp. (Malvaceae) in the Hawaiian Islands
Source: Front Plant Sci. 2024 Mar 1;15:1304078. doi: 10.3389/fpls.2024.1304078 (PMC10941843; doi:10.3389/fpls.2024.1304078)

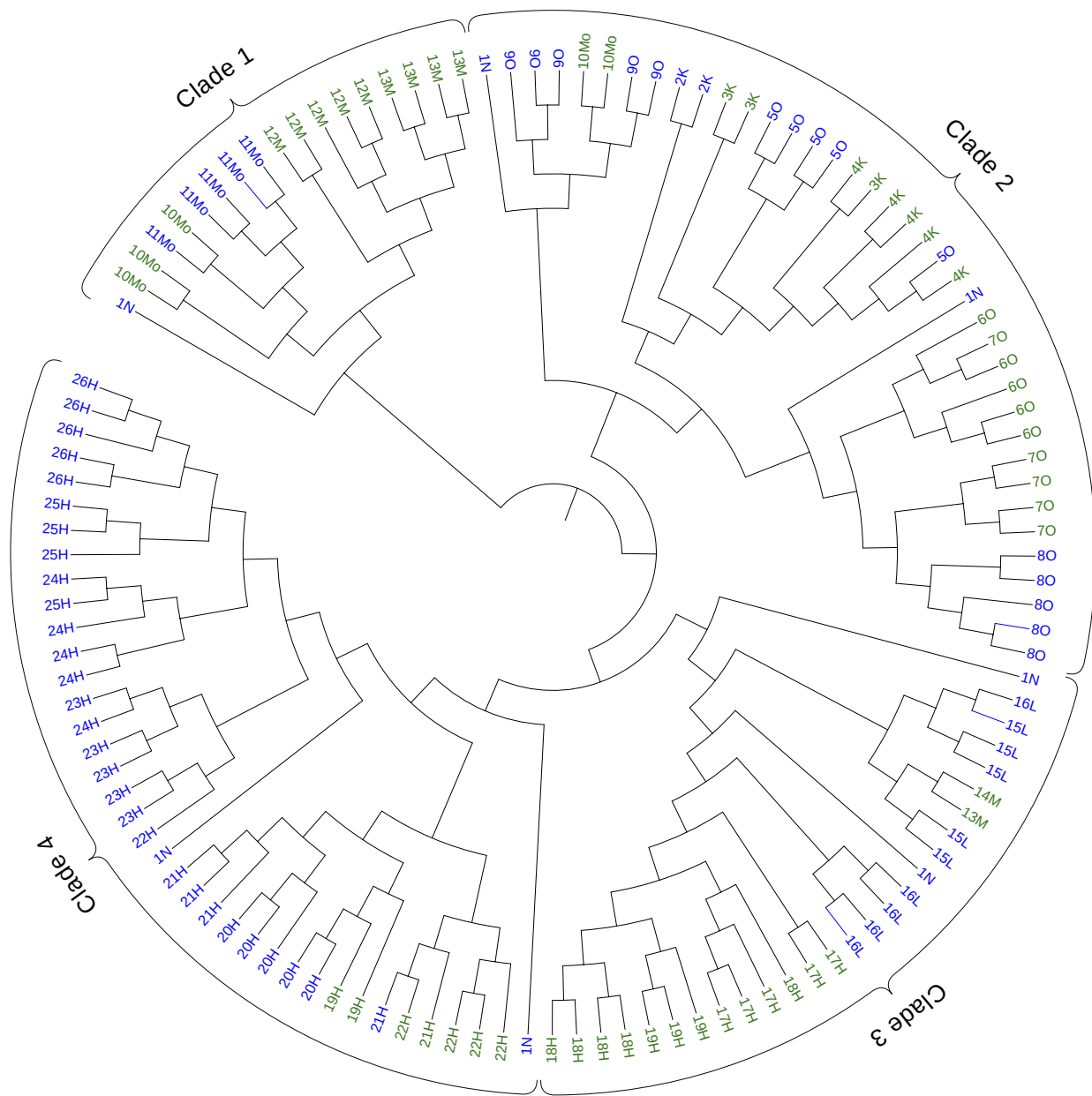

Supplement: Supplementary Figure 1 — Neighbor Joining tree using MIG-seq data for 124 individuals in 26 populations of Sida fallax across the Hawaiian Islands. Island abbreviations: N, Nihoa; K, Kauaʻi; O, Oʻahu; M, Maui; Mo, Molokaʻi; L, Lānaʻi; H, Hawaiʻi. Population numbers correspond to those in Table 1 . Blue color: mountain/inland populations; Green color: beach populations. [file DataSheet_1.zip › Supplementary Figure 1.PDF]

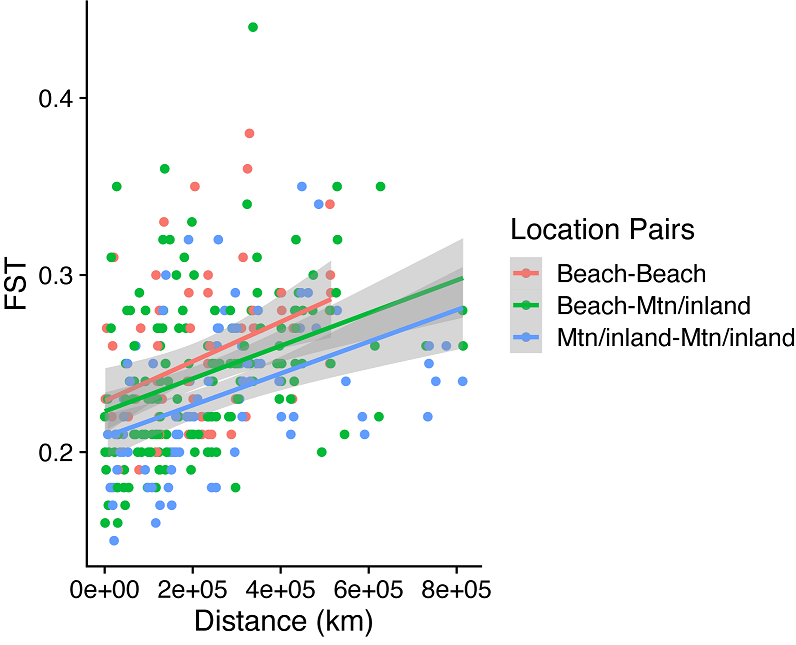

Supplement: Supplementary Figure 1 — Neighbor Joining tree using MIG-seq data for 124 individuals in 26 populations of Sida fallax across the Hawaiian Islands. Island abbreviations: N, Nihoa; K, Kauaʻi; O, Oʻahu; M, Maui; Mo, Molokaʻi; L, Lānaʻi; H, Hawaiʻi. Population numbers correspond to those in Table 1 . Blue color: mountain/inland populations; Green color: beach populations. [file DataSheet_1.zip › Supplementary Figure 2.PNG]
